# Clinical efficacy and safety of somatostatin in the treatment of early postoperative inflammatory small bowel obstruction: A protocol for systematic review and meta analysis

**DOI:** 10.1097/MD.0000000000020288

**Published:** 2020-05-15

**Authors:** Zhongyong Wu, Suibiao Wang, Shaofei Yuan, Ming Lin

**Affiliations:** a Department of Critical Care Medicine, The Second Affiliated Hospital of Hainan Medical University, Longhua District, Haikou; b Department of Critical Care Medicine, Tunchang County People's Hospital of Hainan Province, Tuncheng Town, Tunchang County, Hainan; c Department of Pharmacy, The Second Affiliated Hospital of Baotou Medical College, Baotou, Inner Mongolia, China.

**Keywords:** a protocol for a systematic review and meta-analysis, early postoperative inflammatory small bowel obstruction, postoperative, PRISMA-P, RCTs, somatostatin

## Abstract

**Background:**

As one of the complications after abdominal operation, early postoperative inflammatory small bowel obstruction (EPISBO) is a great trouble for many patients. The use of somatostatin in treating this disease had been widely reported, but its efficacy and safety were controversial. Therefore, the present research carried out a systematic review of the clinical efficacy and safety of somatostatin in treating EPISBO.

**Methods:**

Computer retrieval was conducted in foreign databases (including PubMed, The Cochrane Library, and Embase) and Chinese database (including Sino Med, CNKI, VIP, and WangFang Data), supplementary search for the literatures included was performed, and manual retrieval was performed in abstracts, books, and non-electronic magazines related to the present research to ensure the recall rate. Among all republished relevant experimental studies in Chinese and English from January 1, 1996 to February 1, 2020, randomized controlled trials on the curative efficacy of somatostatin for EPISBO were collected. The evaluation measures included patients’ total effective rate, peristaltic sound recovery time, time of disappearance of abdominal pain, time of first defecation after operation, drainage of gastrointestinal decompression and length of stay after treatment. The literatures were selected by two investigators independently to extract data according to the inclusion and exclusion criteria, Bias Risk Assessment Tool recommended by Cochrane Review Hand book 5.2 was used to assess literature quality, and meta-analysis was carried out by using soft Stata 12.0.

**Results:**

This study will scientifically and effectively analyze the clinical efficacy and safety of somatostatin in the treatment of EPISBO through systematic review and meta-analysis.

**Ethics and dissemination:**

The results of this study will be published in a peer-reviewed SCI journal to provide evidence-based medical evidence for the clinical treatment of early postoperative inflammatory bowel obstruction.

**Protocol and registration:**

Open Science Framework (https://osf.io, OSF), registration number: ryd2g.

## 1 Introduction

Early postoperative inflammatory small bowel obstruction (EPISBO), a very common complication following colorectal operation, often occurs in the early stage after abdominal operation (within 2–3 weeks). The primary cause of EPISBO is a kind of mechanical and dynamic abdominal adhesion of the intestinal tract due to wall edema and exudation because of injury of abdominal operation and post-operative abdominal inflammation, but intestinal strangulation rarely occurs.^[1]^ EPISBO occurs in the early stage after operation, and the major symptom is abdominal distension with or without mild abdominal pain. Adnominal inflammation-induced intensive intestinal adhesion is the major cause of the disease.^[2,3]^

It is believed in the world of surgery that early postoperative intestinal obstruction is a kind of mechanical bowel obstruction. Such mechanical factors as enteroplegia, intestinal twist, abdominal disease, intestinal hematoma, and anastomotic stenosis may result in early postoperative intestinal obstruction. So does abdominal inflammation. At present, most of scholars believe that early postoperative intestinal obstruction involves in repeated abdominal pain and vomit after the recovery of intestinal function with 1 month after operation, as well as imaging evidence of intestinal obstruction.^[4,5]^ Foreign scholars has found that the incidence of postoperative intestinal obstruction is as high as 30%, while postoperative inflammatory intestinal obstruction accounts for about 90% of early inflammatory intestinal obstruction after abdominal surgery.^[6]^

With the continuous rise of abdominal operation rate, the incidence of EPISBO is also improving. If left untreated, intestinal function disorder and a series of other complications such as abdominal infection and peritoneum effusion may be caused, which is not good for patients’ postoperative recovery, increases their pain and prolongs length of stay.^[7]^ Improper treatment measures may lead to intestinal fistula and short-bowel syndrome and even death. Currently, some reported treatment methods include gastrointestinal decompression, oral administration of western medicine, oral administration of traditional Chinese medicine (TCM), TCM enema, acupuncture, moxibustion, etc. However, since EPISBO patients with severe abdominal and intestinal adhesions and inflammation, surgical exposure is very difficult and intestinal damage easily happens.^[8,9,10]^ Therefore, conservative treatment-based first-line treatment has been gradually developed.^[11]^ Considering that conservative treatment has multiple modalities, they have variant efficacy and safety in clinical applications. As one of conservative treatment methods, drug treatment fuels controversy about the efficacy and safety of various drugs among clinicians.

Somatostatin was extracted from hypothalamus by Brazeau et al. in 1973 for the first time. Its full name is growth hormone-inhibiting hormone, a kind of cyclic peptide hormone consisting of 14 amino acids. It is widely distributed in pancreatic D cells and gastrointestinal autonomic nerves and can obviously inhibit the secretion of digestive juice in the gastrointestinal tract. Studies have shown that SS is used in treating EPISBO as it can significantly reduce the accumulation of digestive juice in intestinal canal above the obstructed segment, reduce dilatation and ischemia of intestine due to the accumulation of a large amount of fluid, accelerate the recovery of intestinal wall blood circulation and promote inflammation resolution.^[12]^ In order to explore the effectiveness and efficacy of SS as one of conservative therapy in treating EPISBO, we carried out a systematic review and a meta-analysis of the included randomized controlled trials so as to assess its effectiveness and efficacy in an objective manner.

## 2 Methods

### 2.1 Protocol and registration

Prospective registration of this study has been approved by Open Science Framework (https://osf.io, OSF), registration number: ryd2g.

### 2.2 Ethics and dissemination

Ethical approval is not appropriate for this study. The results of this study will be published in a peer-reviewed SCI journal to provide evidence-based medical evidence for the clinical treatment of early postoperative inflammatory bowel obstruction.

### 2.3 Eligibility criteria

#### 2.3.1 Types of studies

Blinded or non-blinded studies on randomized controlled trials of somatostatin for the treatment of EPISBO in Chinese or English.

#### 2.3.2 Types of participants

EPISBO patients undergoing abdominal surgery, regardless of surgical type or surgical method, were included. Patient data were collected without limiting by age, gender or case source. The diagnostic criteria refer to the diagnostic criteria for early inflammatory bowel obstruction in the literature.^[13,14,15]^

Inclusion Criteria: Inclusion criteria:

1. There is a recent (within 1–3 weeks) history of abdominal surgery, especially a recent history of repeated surgeries and extensive separation of adhesions.
2. The peristalsis of all patients recovered at one time, but some patients had been discharged and had a bowel movement and developed intestinal obstruction symptoms 3 to 7 days later.
3. Abdominal X-ray showed many gas–liquid planes or intestinal pneumatosis; abdominal CT showed intestinal wall thickening, edema, adhesions, intestinal effusion, and pneumatosis of peritoneal exudate.
4. In addition to external hernia, volvulus and anastomotic stenosis caused by factors such as mechanical intestinal obstruction; in addition to external causes of hypokalemia, visceral nerve injury and tumor metastasis caused by intestinal obstruction.

Exclusion Criteria:

1. Non-RCT documents;
2. Medicines not related to this research;
3. Patients whose baseline data are obviously inconsistent;
4. Patients whose inflammatory intestinal obstruction does not occur following abdominal surgery;
5. Case report, review, animal experiment, or important data report are incomplete but the author does not give a reply;
6. The clinical outcomes included into the research do not involve those clinical outcome we want to assess.

#### 2.3.3 Types of interventions

Experimental group: somatostatin combined with conventional treatment (dosage, times of administration, course of disease, interval of medication, course of treatment were not limited). Control group: conventional treatment (dosage, times of administration, course of disease, interval of medication, course of treatment were not limited).

#### 2.3.4 Types of outcome measures

##### 2.3.4.1 Primary outcomes

Evaluation measures were

1. Evaluation measures were patients’ peristaltic sound recovery;
2. Time of disappearance of abdominal pain;
3. Time until first defecation after operation;
4. Gastrointestinal decompression;
5. Total effectiveness rate.

##### 2.3.4.2 Secondary outcomes

1. Length of stay;
2. The occurrence of adverse reactions.
3. Serum inflammatory factors;
4. Incidence of adverse reactions.

### 2.4 Search methods for the identification of studies

#### 2.4.1 Electronic searches

The election procedure of this meta-analysis was performed in strict accordance with Preferred Reporting Items for Systematic Reviews and Meta-Analyses (PRISMA). Computer-based retrieval was conducted in China National Knowledge Infrastructure (CNKI), WanFang Data, PubMed, Cochrane Library, and Embase, so as to collect relevant all republished relevant experimental studies in Chinese and English from January 1, 1996 to February 1, 2020 and follow up on the references of the studies included. Regardless of major result or language, all possibly qualified studies were considered for review. Therefore, manual search was performed by referring to the list of major English papers. Relevant abstracts, books and non-electronic magazines in Chinese and English were searched manually.

#### 2.4.2 Other sources

We will search the references of the selected literature and the data of conference papers that have not been published. We will try our best to collect the data and historical data related to this research.

#### 2.4.3 Search strategy

The keywords used for search are as follows: Chinese keywords used Chinese phonetic alphabet “Shengzhangyisu,” “Shuhouzaoqichanggengzu,” “Shuhouzaoqiyanxingchanggengzu,” and “Changgengzu”; English keywords are “somatostatin,” “somatostatain,” “inflammatory ileus,” “EPISBO,” “early postoperative inflammatory ileus,” etc. The complete PubMed search strategy is summarized in Table 1.

**Table 1:**
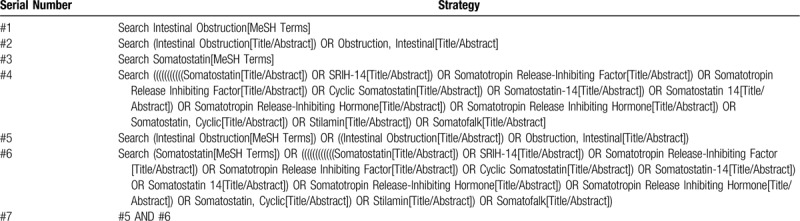
Searching strategy in PubMed.

### 2.5 Data collection and analysis

#### 2.5.1 Data extraction

**Figure 1:**
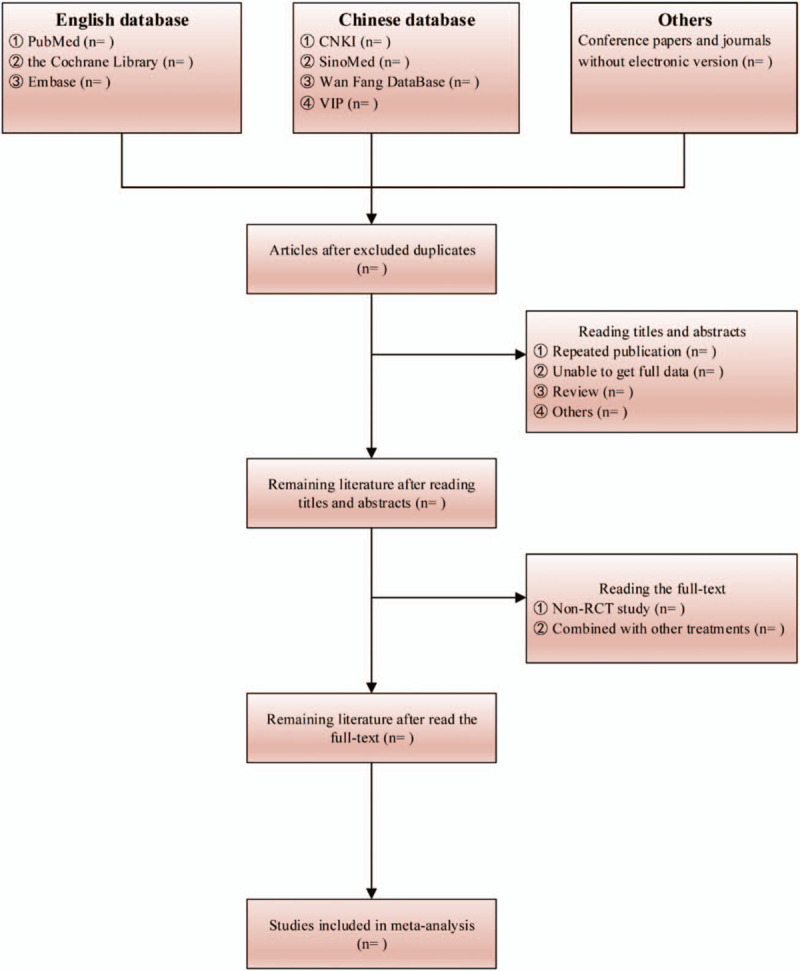
The PRISMA flow chart.

Two evaluators read document titles and abstracts independently and read through them when necessary. Documents were selected according to the pre-determined inclusion and exclusion criteria. Evaluators read through the documents that met the inclusion criteria to make further confirmation. Any document on which the two evaluators has different opinions were discussed by the third evaluator to decide whether to include it. After selecting the documents to be included, we prepared a table of extracted data, including research objects (investigator, origin of study, sample size, age, gender, baseline data, diagnostic criteria, inclusion criteria, and exclusion criteria), research methods (random method and allocation concealment; blinding of patients, intervention provider and result evaluators; number of dropouts and subjects lost to follow-up), intervention (intervention methods, treatment times and follow-up periods of the experimental group and control group) and clinical outcomes (type of outcome and measuring method). The PRISMA flow chart of the study is shown in Figure 1.

#### 2.5.2 Addressing missing data or unclear measurement scales

If any article had no specific description or relevant data were missing, the author was contacted for obtaining further information.

### 2.6 Assessment of heterogeneity

Bias risk assessment tool, which is recommended by Cochrane Review Hand book 5.2, was employed to assess selection bias (stochastic series generation and allocation concealment), implementation bias (blinding of investigators and subjects), measurement bias (blind evaluation of research result), follow-up bias (completeness of result data), report bias (selective report of research results), and other bias.^[16]^ “Low bias risk,” “uncertain bias risk,” and “high bias risk” were used to determine each measure. Low bias risk refers to that the existing bias would not have a great impact on the research result, uncertain bias risk suggests that the existing bias lowers the credibility of the research result, and high bias risk means that the existing bias greatly undermines the credibility of the research result.^[17]^

### 2.7 Data analysis

Statistical software Stata12.0 was used to conduct a meta-analysis of the data collected. Enumeration data were expressed in risk ratio (RR) and continuous variable were expressed in weighted mean difference (WMD) (both confidence interval were 95%). Heterogeneity test (*Q* test) was conducted on each experimental result. *P* > .1 was considered to suggest no heterogeneity; *P* ≤ .1 was considered to reveal heterogeneity. The degree of heterogeneity was expressed by Higgins *I*^2^ (*I*^2^ = (*Q* − df)/*Q*). If there was statistical homogeneity between various research results (*P* > .1, *I*^2^ ≤ 50%), meta-analysis was conducted by using the fixed effect model; if there was statistical heterogeneity between various research results (*P* ≤ .1, *I*^2^ > 50%), meta-analysis was conducted by using the random effects model after eliminating the effect of obvious clinical heterogeneity. Subgroup analysis or sensitivity analysis was performed to process obvious clinical heterogeneity, or only descriptive analysis was provided.

### 2.8 Sensitivity analysis

In order to verify the stability of these results, we carried out sensitivity analysis to eliminate studies having a great impact on the result. No change to the result suggested that the result was stable. In contrast, if there was any change to the result, it was required to carefully analyzes and study the eliminated documents to identify the source(s) of heterogeneity and make a prudent conclusion, or only give a description analysis.

### 2.9 Subgroup analysis and solutions to heterogeneity

For the results with great heterogeneity among the clinical outcomes, subgroup analysis was performed on them according to the information contained in the documents, so as to find out the reasons of enlarged heterogeneity through subgroup analysis. Meanwhile, an appropriate subgroup analysis could reduce the heterogeneity of the results and increase their reliability.

### 2.10 Assessment of reporting bias

Publication bias was assessed when the subgroup included ≥8 studies. The publication bias was checked by either the visual funnel plot or Egger's test.

## 3 Discussion

Early postoperative inflammatory small bowel obstruction is a type of early intestinal obstruction after abdominal operation, accounting for about 20% of intestinal obstruction after abdominal operation. Its major pathological and physiological changes are inflammatory edema, exudation, and adhesion of intestinal wall, leading to a kind of obstruction with both mechanical factor and dynamic factor. It has been universally recognized that early surgical treatment is not suitable for EPISBO, and non-surgical treatment should be the mainstream. Such disease often occurs in:

1. patients with a wide operative range, server injury, and long operative time;
2. patients whose onsite time is mostly within 2 weeks after operation;
3. patients who suffer from obstruction again after early postoperative short gas exhaust, defecation, and food intake, have increasingly severe symptoms and have one of the following four typical clinical characteristics (abdominal pain, abdominal distention, vomiting, cessation of annual gas exhaust, and defecation).

The treatment methods are most basic treatments

1. fasting and water deprivation;
2. sustained effective gastrointestinal decompression^[18]^;
3. spasmolysis and analgesia to relieve patients’ symptoms;
4. correct water, electrolyte, and acid–base balance disturbances;
5. anti-infection combined with other non-surgical therapies.

Drug treatment is one of the non-surgical treatments for such disease. Somatostatin is an effective drug that has been used in clinical. As a common skin hormone, somatostatin is mainly distributed in posterior pituitary, stomach private membrane, pancreatic island, etc. Somatostatin can effectively inhibit gastric acid secretion and peristole, inhibit insulin secretion and growth hormone (GH) secretion and has a certain inhibiting effect on the releases of pepsase and gastrin.^[19]^ Therefore, it has been used in clinic to treat EPISBO. Although many treatment drugs have been developed, they differ a lot in curative efficacy.

Consequently, there are many disputes on the efficacy and safety of these drugs. Taking somatostatin as the research object, this research carried out a systematic evaluation on its efficacy so as to increase sample size, enhance the credibility of studies and provide reliable theoretical evidence for the clinical treatment of EPISBO.

## Author contributions

**Conceptualization:** Ming Lin.

**Data curation:** Zhongyong Wu, Suibiao Wang, Shaofei Yuan.

**Investigation:** Zhongyong Wu, Shaofei Yuan.

**Methodology:** Suibiao Wang, Shaofei Yuan.

**Software:** Zhongyong Wu, Shaofei Yuan.

**Writing – original draft:** Zhongyong Wu, Suibiao Wang, Shaofei Yuan.

**Writing – review & editing:** Ming Lin.
